# Evaluating the Feasibility of Methods of Live Data Collection for Sociability Assessments

**DOI:** 10.1007/s40617-025-01101-9

**Published:** 2025-10-09

**Authors:** Grace E. Sigwanz, Gabriela Salazar, Janelle K. Bacotti, Samuel L. Morris

**Affiliations:** 1 Department of Psychology, University of Miami, Coral Gables, FL USA; 2 Department of Psychology, Louisiana State University, Baton Rouge, LA USA

**Keywords:** Data collection, Discontinuous measurement, Feasibility, Procedural fidelity, Sociability assessments

## Abstract

Sociability assessments may be useful for determining the function of social interaction; however, intensive data collection requirements may limit their clinical feasibility. The purpose of the current studies was to evaluate the feasibility of implementing sociability assessments while collecting discontinuous versus continuous data. We compared procedural fidelity and interobserver agreement across sociability assessment sessions during which implementers collected no data, momentary time sampling data, or duration data in role-play (both studies) and clinical (study 2) contexts. Most participants exhibited relatively high procedural fidelity and interobserver agreement scores remained high across conditions requiring live momentary time sampling and duration data collection. These findings support the feasibility and adoptability of sociability assessments in research and clinical practice. Directions for future research related to sociability assessments as well as general methods of evaluating the feasibility of different data collection procedures are discussed.

**Supplementary Information:**

The online version contains supplementary material available at https://doi.org/10.1007/s40617-025-01101-9.

Social interactions are ubiquitous in the natural environment and their function or the type and degree of influence they exert on behavior is important to consider. Behavior analysts have developed several methods of evaluating the function of social interaction. Perhaps the most fundamental method is a reinforcer assessment (e.g., Morris et al., 2025; Piazza et al., 1999; Smaby et al., 2007). During reinforcer assessments, we deliver social interactions contingent on an arbitrary target response and evaluate the degree to which social interactions support an increase in response rate to determine whether or not social interactions are reinforcing.

More recently, researchers have developed preference assessments for social interaction (e.g., Clay et al., 2013; Nuernberger et al., 2012; Kelly et al., 2014; Morris & Vollmer, 2019; 2020a; 2020b). During preferences assessments, we deliver social interactions contingent on a simple selection or approach response and evaluate the relative occurrence of selection responses to create a hierarchy of preference. Researchers have demonstrated that the social preference assessment hierarchies often correspond to the results of reinforcer assessments (Morris et al., 2023).

The results of test conditions evaluating whether the presentation (e.g., Iwata et al., 1982; Kodak et al., 2007) or removal (e.g., Cengher et al., 2021; Cengher et al., 2022; Harper et al., 2013; Slocum et al., 2021) of social interaction may be the functional reinforcer for challenging behavior are also a method of evaluating the function of social interaction. If challenging behavior occurs at elevated rates in a condition where it produces the addition of social interaction, then we may conclude that social interaction is reinforcing. In contrast, if challenging behavior occurs at elevated rates in a condition where it results in the termination of social interaction, then we may conclude that social interaction is aversive.

Sociability assessments are another method of assessing the function of social interaction that researchers have recently developed and evaluated (e.g., Call et al., 2013; Morris & Vollmer, 2020c; 2021; 2022a; 2022b; Morris & Pizzuto, 2023). One factor that makes sociability assessments unique relative to the methods discussed above is that they concurrently allow for approach and avoidance to social interaction and, as a result, can efficiently delineate whether social interaction functions as a reinforcing, neutral, or aversive stimulus. During sociability assessments, a room is divided in half with identical leisure activities available on each side. The two halves of the assessment only differ in terms of the availability of social interaction. A therapist who delivers generic social interactions and reinforces clear mands for more individualized types of play or interaction is present on one side of the room, which is designated the social side. In contrast, no people or social interaction are available on the other side of the room, which is designated the independent play side. The side on which social interaction is available is systematically manipulated within each session to evaluate whether and how social interaction influences participant’s behavior. The only way participants can consistently maintain access to or avoid social interaction is by altering their time allocation based on changes in the availability of social interaction. Behavior analysts can determine whether social interaction functions as a reinforcing, neutral, or aversive stimulus based on the way participants allocate their time during sociability assessments (Morris & Vollmer, 2021).

Sociability assessments may serve a few important functions in behavior-analytic research and practice. They may be used to evaluate the need for and efficacy of interventions related to improving sociability. For example, sociability assessments could be routinely conducted at the onset of services to screen for indifference to and avoidance of social interaction. Then, when consistent indifference or avoidance is observed, assessment results could provide a baseline with which to evaluate intervention strategies such as incorporating individualized, preferred types of play and social interactions (e.g., Morris & Vollmer, 2022a; Sivaraman & Fahmie, 2018), teaching new communication (e.g., Mattson et al., 2023; Seaver & Bourret, 2020) or play (e.g., Agana et al., 2025; Pane et al., 2022) skills, or improving the function of social stimuli (e.g., Dudley et al., 2019; Jimenez-Gomez et al., 2024; Sainsbury et al., 2024). Using sociability assessments to screen for indifference and avoidance and evaluate corresponding intervention strategies could also help to prevent the development of social avoidance and challenging behaviors reinforced by the termination of social interaction (e.g., Cengher et al., 2022; Slocum et al., 2021). However, if sociability assessments are not feasible to routinely conduct in research and practice, then behavior analysts may not contact or expand upon this potential utility.

Researchers have recently evaluated methods of reducing the complexity and improving the efficiency of sociability assessments (e.g., Morris & Vollmer, 2020c; 2021; 2022a; Morris & Pizzuto, 2023). However, one unaddressed characteristic that may limit feasibility is the number of personnel required to conduct a sociability assessment. In all previous research, sociability assessments have required one therapist whose sole responsibility was implementing the sociability assessment procedures. In most cases, one or two additional research assistants have been present to video record the session and collect data, respectively (e.g., Call et al., 2013). Although using additional personnel to support or assist with assessment implementation is common in research, doing so may often be infeasible in clinical practice. One potential solution could be to use mounted video equipment to record sessions that are conducted in the absence of additional personnel beyond the therapist who implements sociability assessment procedures, which would allow for data to be collected from video recordings after-the-fact. Although this may circumvent personnel limitations, it may double the duration required to complete a sociability assessment and require additional time in the absence of the participant or client with whom the assessment was conducted, making this strategy infeasible in many clinical contexts. Thus, additional research is needed to evaluate methods of data collection that are feasible to use while implementing sociability assessment procedures, which could circumvent prohibitive personnel or time requirements.

To date, the primary dependent variable during sociability assessments has been the duration of social time allocation (Call et al., 2013; Morris & Vollmer, 2020c; 2021; 2022a; 2022b; Morris & Pizzuto, 2023). The feasibility of conducting sociability assessments while collecting duration data has yet to be evaluated. It is unknown if implementers can reliably collect duration data while maintaining high procedural fidelity. If collecting duration data while implementing sociability assessments degrades procedural fidelity, then methods of discontinuous sampling may provide a more feasible alternative that closely approximates duration data. Partial interval recording and momentary time sampling have both been found to provide close approximations^1^ to continuous measures such as frequency and duration (Devine et al., 2011; Harrop & Daniels, 1986; LeBlanc et al., 2020; Rapp et al., 2007; Rapp et al., 2008; Schmidt et al., 2013) and to allow for similar conclusions about assessment (e.g., Gardenier et al., 2004) and intervention (e.g., Meany-Daboul et al., 2007) outcomes. Importantly, researchers have found that the degree of correspondence between methods of discontinuous sampling and continuous measurement was inversely related to the size of the interval used for discontinuous sampling: correspondence tends to decrease as intervals become larger (e.g., Alvero et al., 2008; Gunter et al., 2003).

Given the potential for correspondence between methods of discontinuous sampling and continuous measurement, it is important to consider whether methods of discontinuous sampling are feasible to use, in general, and especially while implementing assessment or intervention procedures (Fiske & Delmolino, 2012; LeBlanc et al., 2015). Previous research suggests that methods of discontinuous sampling can yield high levels of interobserver agreement between two data collectors (Hanley et al., 2007) and that methods of discontinuous sampling that are preferred by data collectors can readily be identified (Hanley et al., 2007; Kolt & Rapp, 2014). Data collectors have been found to display higher levels of interobserver agreement while, and a consistent preference for, using methods of discontinuous sampling with larger intervals (Hanley et al., 2007; Kolt & Rapp, 2014). Interobserver agreement and procedural fidelity are essential measures in applied behavior analysis as they (a) allow for opportunities to provide feedback to data collectors and therapists, (b) permit long-term monitoring of performance trends, and (c) elucidate the extent to which accurate clinical decisions can be made from the data (Vollmer et al., 2008).

To our knowledge, researchers have yet to evaluate the feasibility of implementing any form of assessment or intervention procedures while collecting continuous versus discontinuous data. As a result, the degree to which methods of continuous measurement or discontinuous sampling may be feasible to use while conducting sociability assessments also remains unclear. It is important to evaluate how concurrent data collection and implementation requirements may impact the quality of data collected and the fidelity with which procedures are implemented because behavior analysts are often required to serve this dual role in clinical practice (e.g. Fiske & Delmolino, 2012). Therefore, the purpose of the current studies was to evaluate the feasibility of collecting continuous versus discontinuous data while simultaneously conducting sociability assessments in role-play and clinical contexts.

## General Method

Below, we present the methods and results for two distinct studies, both of which aimed to evaluate the utility and feasibility of different data collection methods for sociability assessments. We evaluated the feasibility of collecting discontinuous and continuous data while conducting sociability assessments with a role-play partner (both studies) or child (study 2 only). Across both studies, we conducted sociability assessments using the same arrangement, procedures, and response definitions. The next section describes methodological components consistent across both studies.

### Arrangement and Procedure

Both studies included sociability assessments conducted in the manner described by Morris and Vollmer (2021). All sociability assessments occurred in a room divided into two identical halves by a line of tape. Tables, chairs, and generic, developmentally appropriate toys were present on each side of the room. We individualized the available toys to meet the needs of the child by removing toys that the child was reported to engage with in an unsafe manner. The implementer did not move the toys during the assessment sessions. However, it is important to note that the child or role-play partner could move toys from one side of the room to the other (see Morris & Vollmer, 2020c for discussion and rationale). Other materials included a timer, device equipped with Countee® for data collection (Slanzi & Fernand, 2024), and a GoPro® to video-record sessions. Countee® is a mobile data collection application that produced a data stream that indicated when the child (study 2) or role-play partner (both studies) was on the social side on a second-by-second basis. We modified the settings of the devices equipped with Countee® to avoid the screen turning off during data collection.

An implementer was always present on one side of the room. The side where the implementer was present was designated as the social side because the implementer provided social interactions whenever the child (study 2) or role-play partner (both studies) was also on that side. The implementer delivered generic, non-individualized social interactions approximately once every 10 s that the child or role-play partner was on the social side (i.e., comparable to a variable interval 10-s schedule). Examples of the generic interactions provided by the implementer included praise-like statements (e.g., “You are playing so nicely!”), descriptions of the child’s or role-play partner’s behavior and environment (e.g., “That’s a blue ball!”), and child- or role-play partner-directed play (e.g., coloring together). Examples of nongeneric interactions that the implementer refrained from providing included therapist-directed play (e.g., “Let’s play with the blocks!”), questions (e.g., “What is your favorite color?”), and any interactions tailored to the child’s preferences that were not clearly requested (e.g., providing tickles in the absence of a mand for tickles). The implementer only delivered preferred, individualized social interactions contingent on clear mands, and did not prompt or direct the child or role-play partner’s communication, play, or movement. The implementer reinforced all mands for individualized play or social interaction emitted on the social side (i.e., a fixed ratio 1 schedule), but this did not alter the schedule of generic social interactions described above. Examples of clear mands included specific vocal requests (e.g., “Up”) and gestures (e.g., hand leading the therapist to hold a ball), whereas examples of unclear mands included all nonspecific or unintelligible vocalizations and gestures (e.g., tapping the therapist). The side where the implementer was not present was designated the independent play side^2^ because no social interactions were available when the child or role-play partner was present on that side of the room. When the child or role-play partner was on the independent play side, the implementer did not deliver social interactions and maintained a neutral gaze and posture. The child and role-play partners could move freely about the room and could instantly access social interaction on the social side and leisure items on either side of the room (i.e., there was no changeover delay). The implementer switched between the two sides of the room every 2 min during each 8-min session to systematically manipulate the availability of social interactions. The implementer always signaled that they were switching sides by making a statement such as, “I’m going to play over here now.” Therefore, the only way the child or role-play partner could consistently access or avoid social interaction was by altering their social time allocation based on changes in the availability of social interaction^3^.

### Response Definitions

In both studies, the primary dependent measure was the duration of *social time allocation*, which is consistent with Morris and Vollmer (2021). As described in Morris and Vollmer, a child or role-play partner was recorded as being on the social side when both of their feet were on the same side of the room as the implementer, using a 0-s onset and offset. The child or role-play partner was recorded as being on one side of the room until they met criteria for being on the other side. That is, if the child or role-play partner had one foot on both sides of the line, or both feet on top of the line, then we recorded them as being on the side they were on previously until they met criteria for being on the other side of the room.

## Study 1: Evaluating Feasibility in a Role-Play Context

Study 1 evaluated the feasibility of implementing sociability assessments with a role-play partner while collecting no data, momentary time sampling, or duration data. We selected momentary time sampling as the discontinuous measure to be evaluated on the basis of a retrospective analysis of data sets from Morris and Vollmer (2021) comparing partial interval recording, whole interval recording, and momentary time sampling to duration data. Results suggested that momentary time sampling produced the closest approximation to duration on the social side, which replicated the findings of previous research in this area (e.g., Harrop & Daniels, 1986; LeBlanc et al., 2020; Meany-Daboul et al., 2007). Supporting Information A provides a complete description of the procedures and findings of the retrospective reanalysis. Study 1 consisted of two parts. First, we trained undergraduate research assistants to implement and collect data for sociability assessments. Second, we conducted a recording feasibility evaluation which compared implementer’s procedural fidelity and interobserver agreement across conditions with different data collection requirements (i.e., no live data collection, discontinuous measurement [momentary time sampling], and continuous measurement [duration]).

### Participants, Setting, & Materials

We recruited seven undergraduate students to participate in this study: Beth, Carol, Ernest, Kennedy, Lonnie, Meghan, Victoria. All participants expressed interest in gaining research experience related to conducting behavioral assessments and interventions with children with developmental disabilities and provided informed consent to participate in this study. All participants were enrolled in a course designed to provide undergraduate students with hands-on experience and training related to conducting psychological research. Five participants (all but Kennedy and Meghan) were also enrolled in an introductory course on applied behavior analysis. One participant (Kennedy) worked as a Registered Behavior Technician at a local clinic and had considerable experience collecting behavioral data. All other participants reported that they had no experience collecting behavioral data and all participants were unfamiliar with sociability assessments. Table 1 includes the participants’ demographic information, major, and undergraduate student status. Participants completed up to three sociability recording feasibility evaluation sessions per day, two to three days per week, with at least a 10-min break between sessions.

**Table 1:** Summary of study 1 participant demographics and education

Participants	Age (years)	Sex	Race or ethnicity	Major	College enrollment status
Beth	23	Female	White	Psychology	Senior
Carol	20	Female	White	Psychology	Junior
Ernest	21	Male	White	Psychology	Sophomore
Kennedy	22	Female	White	Psychology	Sophomore
Lonnie	20	Male	White	Psychology	Junior
Meghan	19	Female	White	Psychology	Junior
Victoria	22	Female	White	Psychology	Senior

We completed behavioral skills training and the sociability recording feasibility evaluation sessions in a 3.45 by 2.68 m room constructed out of moveable room dividers. We arranged the sociability assessment room and included the same materials as described in the “General Method” section. During the data collection training and the duration condition, participants had access to a device equipped with Countee®, which included a template set to 480 s and included a social side duration key to collect social time allocation data. We implemented momentary time sampling with 30-s intervals. We anticipated this interval duration would be large enough to improve feasibility while remaining small enough to allow for correspondence with duration data (see Supporting Information A). During the momentary time sampling condition, participants had access to a pencil, clipboard, timer, and paper datasheet that provided the start and end time for each 30-s interval across rows and a column for indicating whether the role-play partner was on the social or independent play side at the end of each interval.

### Design

In the sociability recording feasibility evaluation, we used a multielement design to evaluate how different data collection requirements influenced the feasibility of conducting sociability assessments while collecting live data. Specifically, we presented the no live data collection, momentary time sampling, and duration conditions in rapid alternation. We evaluated (a) the procedural fidelity with which participants conducted sociability assessments across all conditions and (b) participants’ interobserver agreement with a trained observer during conditions requiring data collection (i.e., momentary time sampling, duration). We rapidly alternated between these three conditions to minimize the likelihood that practice or exposure effects differentially influenced one condition over another and could constitute a confounding explanation for differentiation between conditions. We implemented conditions in a consistent order to facilitate detection of sequence effects (i.e., rather than randomizing condition order, which would obscure such effects; Sidman, 1960). For most participants, we implemented conditions in the following order: no data collection, momentary time sampling, and duration. We selected this order in an effort to facilitate higher levels of performance during the initial exposure to conditions requiring data collection, which we anticipated would be more difficult.

### Procedures

#### Pre-experimental Procedures

We completed multiple training steps before progressing to the sociability recording feasibility evaluation. First, we assigned and discussed the Morris and Vollmer (2021) article. Next, we trained participants to collect duration data until they met 96% interobserver agreement and provided participants with instructions on how to collect momentary time sampling. We selected 96% interobserver agreement as our training criteria because it is a more stringent training criterion than more common requirements of 80% or 90% but still allowed for disagreement on up to two intervals out of the 48 10-s intervals in an 8-min session. Finally, we completed behavioral skills training with participants to practice implementing sociability assessments until they met the mastery criteria of two consecutive rehearsals without error, which signaled that we could commence the study. The exact number of rehearsals required was not documented for participants in study 1, but all participants met mastery criteria in five or fewer rehearsal sessions. We completed these training steps before participants progressed to the sociability recording feasibility evaluation to control for the history of accurate implementation and data collection during sociability assessments. These preliminary steps corresponded with competency-based training standards supported within applied behavior analysis (Behavior Analyst Certification Board, 2018; Parsons et al., 2012) to promote high procedural fidelity and accurate data collection before evaluating the isolated effects of a data collection requirement Supporting Information B provides a more detailed description of the study 1 pre-experimental training procedures that preceded our experimental recording feasibility evaluation described below.

#### Sociability Recording Feasibility Evaluation

The evaluation consisted of three conditions: no data collection, momentary time sampling, and duration. Across all conditions, participants implemented the sociability assessment with a role-play partner, according to the procedures described in the “General Method” section. During the no data condition, there was no data collection requirement; participants only implemented sociability assessment procedures. During the momentary time sampling condition, the participants collected momentary time sampling data while implementing the sociability assessment procedures. During the duration condition, the participants collected duration data while implementing the sociability assessment procedures. Participants only collected data on social time allocation as this was the dependent measure required to delineate whether social interaction was reinforcing, neutral, or aversive in Morris and Vollmer (2021). Additionally, we conducted different conditions in a consistent sequence for each participant (e.g., no data collection, momentary time sampling, duration). Before each session, we instructed participants that they would collect no data, momentary time sampling, or duration data. For the sessions in the momentary time sampling condition, participants received a paper datasheet and pen to score social time allocation as well as instructions for how to use the data sheet and when to record “social” or “alone” (i.e., independent play) side. Participants also received a timer to signal the end of each 30-s interval. For sessions in the duration condition, participants received a device equipped with Countee® to score social time allocation using duration as well as instructions for how to use the application to record duration on the social side.

Participants completed contrived, 8-min sessions with a role-play partner. We used a role-play partner, instead of a child who may benefit from participating in a sociability assessment, to control the difficulty of implementation. During each session, the role-play partner spent at least 60% of the time on the social side, switched sides at least 20 times (e.g., 2.5 times per min), and emitted at least eight clear mands for specific types of play or social interactions (e.g., “stack the blocks with me”). Although sociability assessment procedures usually specify that the therapist will reinforce mands for movement (e.g., “leave me alone” or “come play over here”), these have rarely been observed in the research conducted to date. As a result, the role play partner did not mand for the participant to switch sides. We completed three or four series of the three conditions with each participant.

#### Preference and Feasibility Ratings

We assessed social validity by obtaining verbal reports of participants’ preference and feasibility ratings for collecting data and implementing sociability assessments, respectively. After completing the sociability recording feasibility evaluation, we asked participants, “If you routinely conducted assessments of sociability, would you rather (a) not collect data during the assessment and collect data after each assessment from a video recording, (b) collect momentary time sampling data during each assessment, or (c) collect continuous duration data during each assessment?” We also asked participants to rate the feasibility of conducting sociability assessments in each condition (i.e., no data collection, momentary time sampling, duration) on a scale from one to five, stating, “Please rate the difficulty or feasibility of each method of conducting assessments of sociability from one (i.e., very easy/feasible) to five (i.e., very difficult/infeasible).”

## Dependent Measures, Interobserver Agreement, and Procedural Fidelity

### Participant’s Dependent Measures

Two trained observers collected data during all sessions of the sociability recording feasibility evaluation. The dependent measures included the participant’s procedural fidelity in implementing sociability assessments and interobserver agreement with trained observers. We assessed *procedural fidelity* by segmenting each session into 10-s intervals and dividing the intervals without an error by the total intervals (48) and multiplying the quotient by 100. This method allowed us to capture moment-to-moment errors in implementation that could occur repeatedly throughout the session and evaluate the degree to which participants consistently implemented sociability assessment procedures with fidelity. *Procedural fidelity errors* were generally related to the timing or signaling of switches and delivery of social interactions. We recorded an error if the implementer did not switch sides of the room within 5 s of or provide a vocal signal for a scheduled switch. On the social side, we recorded an error if the implementer delivered nongeneric vocal interactions, prompted play that was not initiated by the role-play partner, did not deliver generic interactions during a full interval, did not engage in parallel play when the role-play partner displayed item engagement for a full interval, did not engage in play directed by the role-play partner, or did not reinforce clear mands within the interval that they occurred. We compared the percentage of 10-s intervals with no errors across individual sessions of each condition and the mean across conditions and participants.

We assessed *interobserver agreement* by using the proportional agreement method for duration and interval (i.e., occurrence, nonoccurrence) agreement method for momentary time sampling data. We compared the participant’s live data collection to that of a trained observer who did not conduct the sociability assessment. We calculated proportional interobserver agreement by dividing the session into 10-s intervals and comparing each observer’s data on an interval-by-interval basis. Within each interval, we divided the smaller number of seconds on the social side by the larger number of seconds on the social side to obtain a proportion. The sum of the proportions for each interval was divided by the total number of intervals and then multiplied by 100. We calculated interval-by-interval interobserver agreement for momentary time sampling by dividing the number of 30-s intervals with agreement (i.e., both observers scoring social side at the end of the interval) by the total intervals and multiplying the resulting quotient by 100. We compared the interobserver agreement across individual sessions that required data collection (i.e. momentary time sampling and duration conditions) and the mean across conditions and participants.

### Trained Observers’ Interobserver Agreement

As described above, interobserver agreement was one of the primary dependent measures, which we assessed by comparing data collected by participants and a trained observer. However, because the trained primary observer served as the “standard” or point of comparison for participants, we also assessed interobserver agreement for data that they collected. Therefore, a secondary observer independently collected data on the role-play partner’s duration on the social side from video recordings for one randomly selected session from each condition for each participant (i.e., 25 or 33% of session in each condition). We calculated proportional interobserver agreement for duration on the social side, which had a mean of 98% (range, 96% to 99%) across participants.

Procedural fidelity was another primary dependent measure. As described above, we calculated the percentage of 10-s intervals without a procedural fidelity error for each session. A trained primary observer collected data for each session via a live video feed. In order to evaluate interobserver agreement on procedural fidelity data, a secondary observer independently collected procedural fidelity data from video recordings for one randomly selected session from each condition for each participant (i.e., 25 or 33% of session in each condition). We calculated interval-by-interval interobserver agreement for the occurrence and nonoccurrence of procedural fidelity errors. Each session was divided into 10-s intervals, and we compared the primary and secondary observer’s data on an interval-by-interval basis. For each interval, we scored an agreement if both observers indicated that a procedural fidelity error did or did not occur. The number of intervals with agreement was divided by the number of intervals and multiplied by 100. Mean interobserver agreement was 95% (range, 92% to 98%) for procedural fidelity across participants.

### Role-Play Partners’ Procedural Fidelity

In addition to evaluating participants’ procedural fidelity implementing and collecting data during sociability assessments, we evaluated the procedural fidelity of the role-play partner’s social time allocation, social and avoidant switches, and clear mands for play or social interaction during sociability recording feasibility evaluation sessions. We instructed the role-play partner to spend at least 60% of the session on the social side, switch sides at least 20 times for each session, and emit at least eight clear mands for individualized play or interaction for session to control for the difficulty of conducting the assessment and collecting data across conditions. The role-play partner met or exceeded the targeted percentage of session on the social side, number of switches, and number of mands for 75 out of 78 sessions conducted across all conditions and all participants. The exceptions included Ernest’s second momentary time sampling session in which the role-play partner only switched sides 17 times, Victoria’s third no data session in which the role-play partner only emitted seven clear mands, and Lonnie’s first duration session in which the role-play partner only switched sides 18 times.

## Study 1 Results and Discussion

**Fig. 1:**
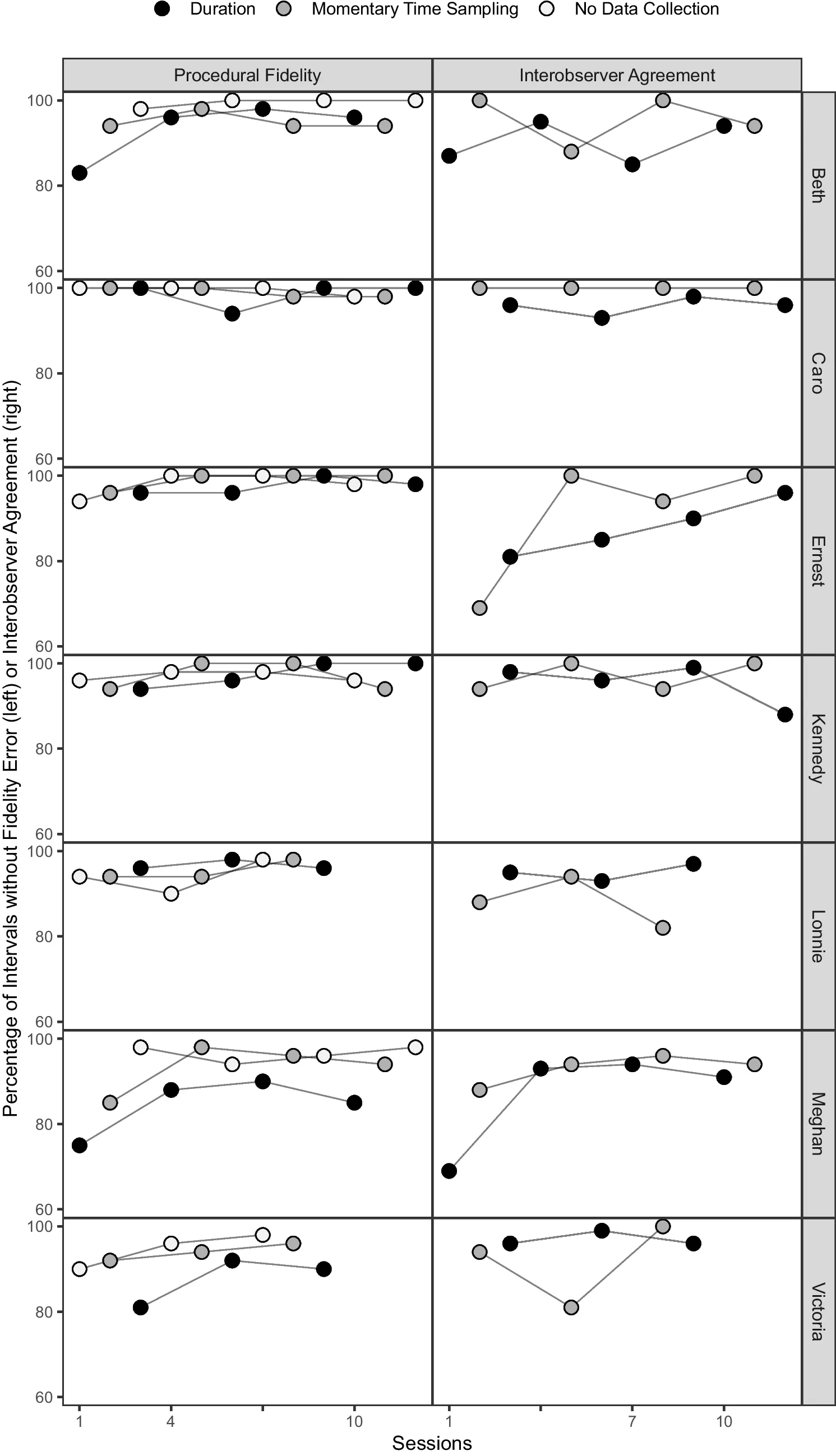
Study 1 participants’ procedural fidelity and interobserver agreement results

**Fig. 2:**
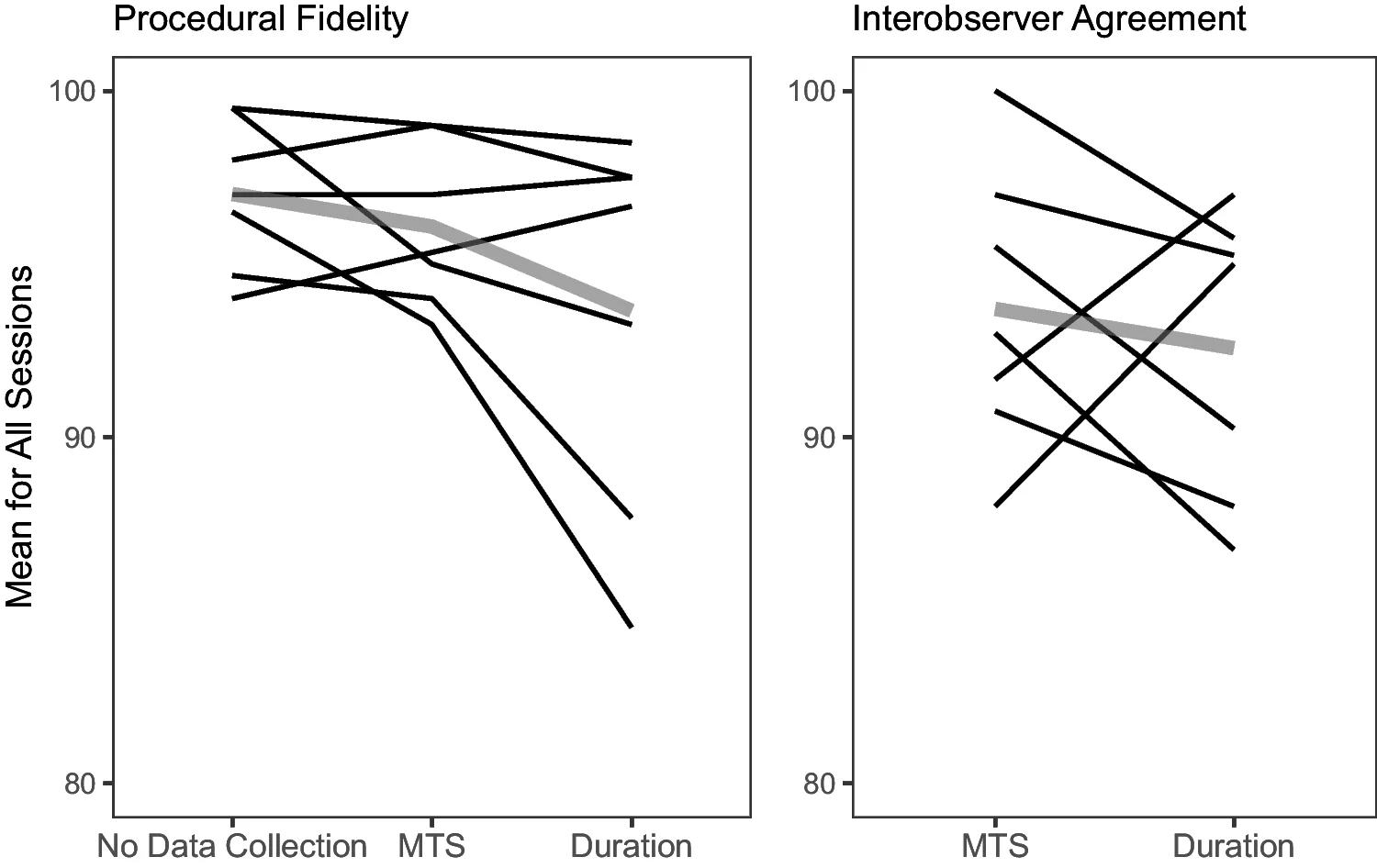
Study 1 participants’ mean procedural fidelity and interobserver agreement results. *Note*. *MTS* Momentary time sampling

Figures 1 and 2 show participants’ procedural fidelity and interobserver agreement data from the recording feasibility evaluation in study 1. The y-axis on both figures does not begin at zero to facilitate visual inspection of variability within and across conditions. The left column of Fig. 1 shows the procedural fidelity for each participant across sessions, with each data point color indicating a different condition within the multielement design (i.e., black, gray, and white for duration, momentary times sampling, and no data, respectively). Four participants (Carol, Ernest, Kennedy, and Lonnie) exhibited minimal (i.e., no more than a 2.67% difference) or no difference in procedural fidelity across no data collection, momentary time sampling, and duration conditions. Three participants (Beth, Meghan, and Victoria) exhibited slightly but consistently higher procedural fidelity during the no data condition compared to the duration condition. During the momentary time sampling condition, these participants’ procedural fidelity was either similar to or slightly lower than the no data condition. The right panel of Fig. 1 shows the interobserver agreement data for each participant across sessions. Scores were high across conditions (i.e., most sessions above 80%); however, most participants (Meghan, Ernest, Carol, and Beth) consistently obtained relatively higher interobserver agreement scores for momentary time sampling. Conversely, two participants (Victoria and Lonnie) obtained higher interobserver agreement scores for duration data. Additionally, one participant (Kennedy) obtained high, undifferentiated interobserver agreement scores for both momentary time sampling and duration. Note that Ernest’s interobserver agreement for duration data maintained an increasing trend across sessions, which may indicate that repeated exposure to or practice with this type of data collection improved interobserver agreement.

Figure 2 shows the mean procedural fidelity (left panel) and interobserver agreement (right panel) for each condition. Each thin black line displays a single participant’s mean procedural fidelity or interobserver agreement across conditions, whereas the thicker gray line displays the mean procedural fidelity or interobserver agreement across all participants during each condition. Mean procedural fidelity scores remained relatively high across conditions, with slightly higher performance during the no data collection and momentary time sampling conditions compared to duration. Interobserver agreement scores also remained high across conditions, with the mean slightly higher in the momentary time sampling condition. However, the outcome obtained varied across participants. Five participants had higher interobserver agreement during the momentary time sampling condition, whereas two participants clearly had higher agreement during the duration condition. Overall, both procedural fidelity and interobserver agreement scores remained relatively high across all conditions (i.e., most sessions above 80%).

Table 2 shows all participants’ reported data collection preferences and their feasibility ratings for each condition. Two participants (Ernest and Victoria) preferred no live data collection, three participants (Beth, Carol, and Kennedy) preferred momentary time sampling, and two participants (Lonnie and Meghan) preferred duration. On a scale of 1 to 5, the mean feasibility ratings were 1.14 (range, 1 to 2) for no live data collection, 2.43 (range, 1 to 4) for momentary time sampling, and 2.86 (range, 2 to 4) for duration. For some participants, preference of data collection method aligned with responding in the respective condition. For example, Lonnie preferred duration data collection and had higher interobserver agreement during that condition. However, such alignment was absent for other participants. Victoria also had higher interobserver agreement for duration, yet she preferred no live data collection. These inconsistencies suggest additional research on methods of live data collection is needed to further investigate and clarify the relation between preference and performance.

**Table 2:** Study 1 participants’ data collection preference and feasibility ratings

		Rating		
Participants	Preferred data collection method	No Data	MTS	Duration
Beth	MTS	1	2	4
Carol	MTS	1	1	2
Ernest	No Data	1	3	4
Kennedy	MTS	1	2	3
Lonnie	Duration	2	4	2
Meghan	Duration	1	2	2
Victoria	No Data	1	3	3

*MTS* Momentary time sampling

Study 1 demonstrated that it may be feasible to collect accurate momentary time sampling or duration data while implementing sociability assessment procedures with a high degree of procedural fidelity. However, four limitations should be noted. First, participants received extensive training on how to collect duration data but were not formally trained on how to collect momentary time sampling data prior to the start of the recording feasibility evaluation. Second, the modality of data collection differed between the duration (digital) and momentary time sampling (paper and pen) conditions and, anecdotally, some participants reported that they would have preferred collecting momentary time sampling data using a digital data system. Third, in study 1, we instructed the role-play partner to allocate a minimum amount of time to the social side, display a minimum number of switches, and emit a minimum number of mands. Although the role-play partner was generally successful in meeting these minimum requirements, only specifying minimum values may have allowed unnecessary heterogeneity in the actual amount of time spent on the social side and number of switches or mands. Fourth, we conducted the recording feasibility evaluation in study 1 entirely in a role-play context, so it remains unclear whether and how differences in procedural fidelity and interobserver agreement would generalize to sociability assessments conducted with individuals from clinical populations. Therefore, the goal of study 2 was to replicate and extend study 1 by addressing the limitations described above. Specifically, in study 2, we (a) formally trained participants to collect momentary time sampling and duration data, (b) incorporated a digital data system for both methods of data collection, (c) provided the role-play partner with discreet text-based coaching related to their time allocation, switching, and mands, and (d) conducted recording feasibility evaluation sessions with a child with autism for whom the sociability assessment was clinically indicated as well as a role-play partner.

## Study 2: Evaluating Feasibility in a Clinical Context

### Participants, Setting, and Materials

We recruited four undergraduate students (Erica, Liz, Margot, Vivian) enrolled for research credits whose schedule aligned with the children enrolled in a clinical research program, all of whom provided informed consent to participate in this study. Participants had no prior coursework in applied behavior analysis, except for a 40-hr Registered Behavior Technician online course. Additionally, participants had limited experience with continuous or discontinuous data collection and no experience using Countee® or implementing sociability assessments. Table 3 includes the participants’ demographics, major, and undergraduate student status. Participants completed sessions with two children enrolled in a clinical research program whose caregivers provided research consent for participating in assessments during their admission. We recruited these children because the onset of their services (i.e., when we would typically conduct an initial sociability assessment) and schedule of weekly appointments were well aligned with the beginning of study 2 and participants availability. These children were 5 and 7 years old, on the autism spectrum, minimally verbal (i.e., limited to no echoics or vocal-verbal behavior), and engaged in aggression (client A) or self-injury (clients A and B). Participants completed up to nine sociability recording feasibility evaluation sessions per day, two to three days per week.

**Table 3:** Summary of study 2 participant demographics and education

Participants	Age (years)	Sex	Race or ethnicity	Major	College enrollment status
Liz	19	Female	Black and White	Philosophy & Math	Sophomore
Erica	20	Female	Latine	Psychology	Junior
Margot	21	Female	White	Psychology & Criminology	Senior
Vivian	21	Female	Latine	Psychology	Senior

We completed behavioral skills training and sociability recording feasibility evaluation sessions in a 2.9 by 2.3 m room with an observation window. We arranged the sociability assessment room and included the same materials as described in the “General Method” section and study 1, except data collection training and sessions only involved the use of a device with Countee®. The duration Countee® template was preset identical to study 1. The momentary time sampling Countee® template was preset to 510 s (8.5-min) and included “social” or “alone” (i.e., independent play) side frequency keys. Participants had the opportunity to reference the running timer on Countee® to score which side the participant was on at the end of every 30-s interval. As with the timer in study 1, the Countee® session clock served as the sole signal to the participants to collect data at the end of each interval. We instructed participants to preset the momentary time sampling Countee® template to 510 s to maintain a 30-s opportunity to score all intervals within a session (i.e., “adding” 30 s prevented Countee® from ending the session before the participant had the opportunity to score the final interval).

### Design

We replicated the multielement design from study 1, with the exception of adding child probes before and after role-play sessions. First, following behavioral skills training, participants completed two series (i.e., six sessions) of the sociability recording feasibility evaluation with a child. We limited these initial child sessions to two series to minimize the child’s potential exposure to low procedural fidelity. Then, participants progressed to completing at least three series with a role-play partner. Finally, participants returned to completing sessions with a child for at least three series to evaluate the effects that contrived, role-play sessions had on procedural fidelity and interobserver agreement when collecting data while implementing a sociability assessment with a child. As in study 1, we also obtained participants’ preference and feasibility ratings after they completed the sociability recording feasibility evaluation.

### Procedures

#### Pre-experimental Procedures

As described for study 1, participants progressed from the pre-experimental procedures (i.e., reading and discussing Morris and Vollmer [2021], data collection training, and behavioral skills training) to the sociability recording feasibility evaluation. As in study 1, the study could commence following behavioral skills training in which participants meet the mastery criteria of two consecutive rehearsals without error. It took participants 3 to 4 rehearsals to meet this criterion. One change for study 2 was that participants received competency-based training for momentary time sampling as well as duration data collection. Supporting Information B provides a detailed description of the study 2 pre-experimental training procedures that preceded the recording feasibility evaluation.

#### Sociability Recording Feasibility Evaluation

The sociability recording feasibility evaluation with a child and role-play partner constituted the experimental procedures of study 2. As described for study 1, each series involved instructing participants to implement sociability assessment sessions while collecting no data, momentary time sampling, or duration data. Participants completed sociability assessment sessions with a child before and after completing at least three series of sessions with a role-play partner. As described in Study 1, the role-play partner distributed their social time allocation, programmatically switched sides, and emitted clear mands for play or social interaction. To ensure and equate the difficulty of conducting the assessments during role-play partner sessions, the role-play partner received discreet text-based coaching via their mobile device to follow one of three scripts, each of which involved spending at least 60% of the session on the social side, switching sides at least 13 times, and emitting eight clear mands for individualized play or interaction during each session. Each participant completed sociability assessment sessions with one (Liz, Erica) or two (Margot, Vivian) children. Child data and the sociability assessment outcomes during each participant’s sessions are available for review as Supporting Information C.

#### Preference and Feasibility Ratings

We completed the same questionnaire described in study 1 to assess social validity by obtaining reports of participants’ preference and feasibility ratings for collecting data and implementing sociability assessments.

### Dependent Measures, Interobserver Agreement, and Procedural Fidelity

#### Participant’s Dependent Measures

As described in study 1, we included the same dependent measures: *procedural fidelity* and *interobserver agreement*. We calculated the percentage of intervals without procedural fidelity errors for each session of each condition for each participant and summarized these data across conditions and across participants. We also assessed the percentage of procedural fidelity errors that occurred when the child was on the social versus independent play side during the child-probe sessions of the sociability recording feasibility evaluation, which may be found in Supporting Information D.

We calculated interobserver agreement on social time allocation using the same proportional agreement and interval-by-interval agreement methods described in study 1 for duration and momentary time sampling data, respectively. We evaluated interobserver agreement for each session of each condition for each participant, and we summarized these data across conditions and across participants.

#### Trained Observers’ Interobserver Agreement

Interobserver agreement was one of the primary dependent measures. As described in study 1, we calculated interobserver agreement by comparing data collected by participants to data collected by a trained primary observer. Similar to study 1, an independent secondary observer also collected data on the role-play partner’s and child’s duration on the social side by reviewing 33% of video-recorded sessions that occurred across conditions and phases for each participant. We randomly selected these sessions, with the caveat that we ensured at least one session from each condition and phase was included. We also assessed interobserver agreement by comparing the primary and a secondary observer’s data using the same calculations for proportional agreement described in study 1. We obtained a mean interobserver agreement of 98.75% (range, 87.5% to 100%) for duration on the social side. Procedural fidelity was also a primary dependent measure. We calculated interobserver agreement on procedural fidelity identically to study 1. Across participants, mean interobserver agreement on procedural fidelity was 98.83% (range, 95.83% to 100%) during initial child sessions, 99.13% (range, 95.83% to 100%) during role-play sessions, and 97.75% (range, 87.5% to 100%) during the reversal to child sessions.

#### Role-Plays Partners’ Procedural Fidelity

As described in study 1, we evaluated the procedural fidelity of the role-play partner’s social time allocation, social and avoidant switches, and clear mands for play or social interaction during sociability recording feasibility evaluation sessions. However, in study 2 the role-play partner received discreet text-based coaching related to their time allocation, switching, and mands rather than simply being given instructions for the target values of each measure as in study 1. The role-play partner met or exceeded the target percentage of session on the social side and number of mands for all sessions conducted across all conditions and participants. However, the role-play partner completed one fewer switch than the minimum (i.e., 12 instead of 13) during four sessions (i.e., 32 out of 36 sessions) but adhered to the scripted switch schedule for all other sessions.

## Study 2 Results and Discussion

**Fig. 3:**
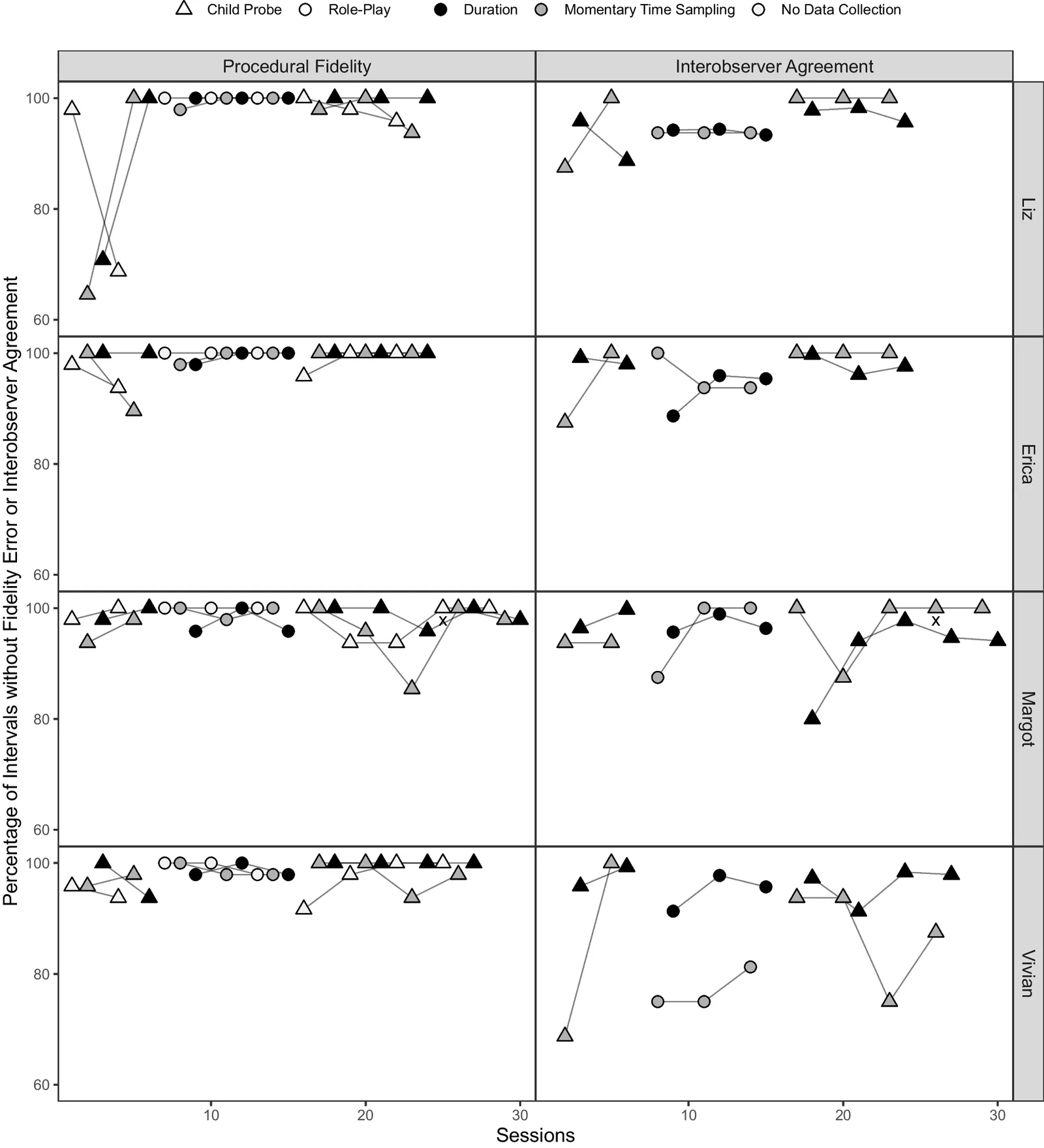
Study 2 participants’ procedural fidelity and interobserver agreement results. *Note*: “x” indicates the session at which Margot began only implementing child probe sessions with Client B, due to aggression observed during sessions with Client A

**Fig. 4:**
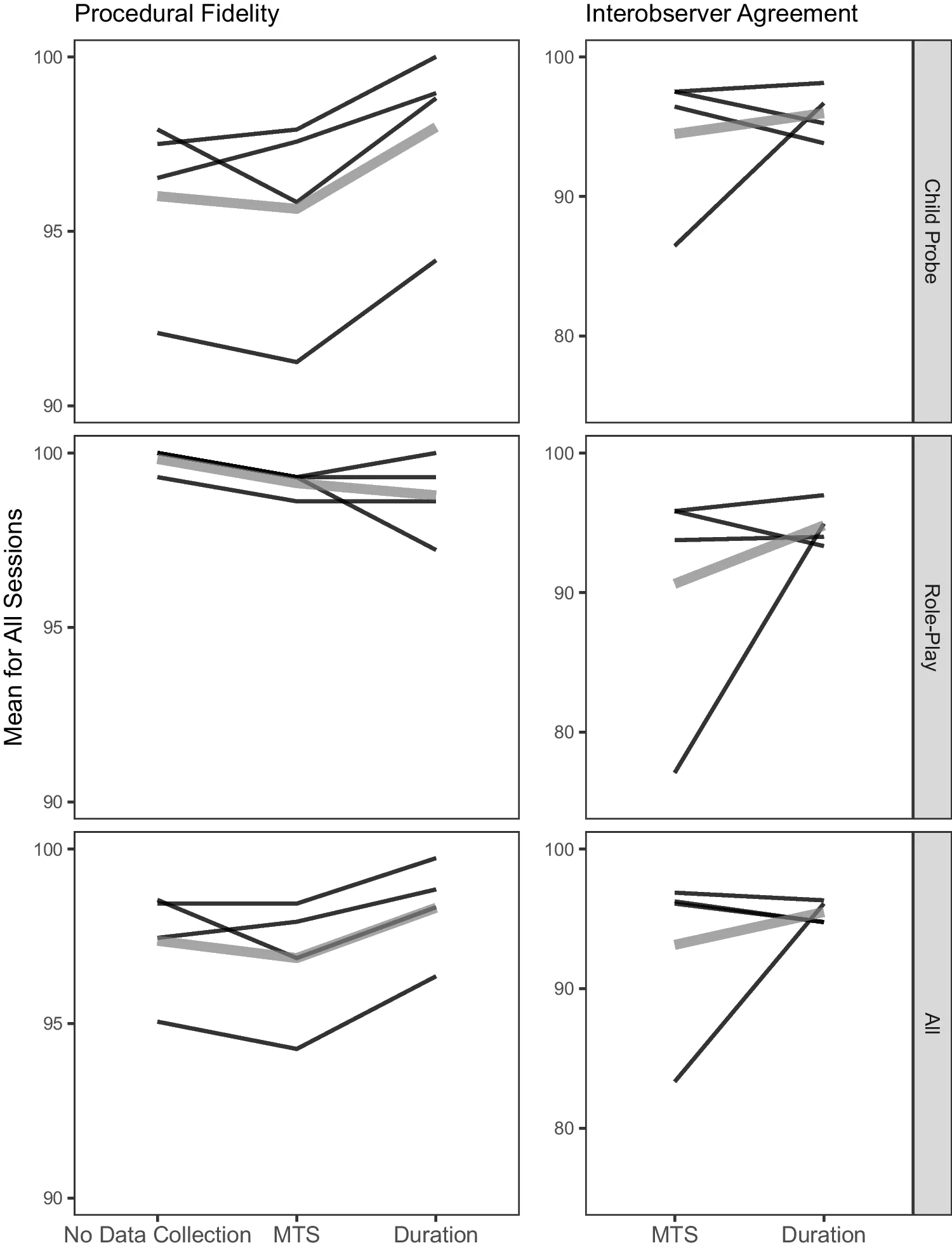
Study 2 participants’ mean procedural fidelity and interobserver agreement results. *Note*. *MTS* Momentary time sampling

Figures 3 and 4 show the results of the sociability recording feasibility evaluation, using similar graphing conventions as Figs. 1 and 2, respectively. The left column of Fig. 3 shows the procedural fidelity for each participant across sessions. Triangles represent sessions completed with a child, whereas circles represent sessions completed with a role-play partner. All participants generally exhibited high, stable procedural fidelity during all phases. However, relative to role-play partner sessions, we sometimes obtained more variability and lower procedural integrity values during child sessions. Margot exhibited temporary decreases during the reversal to child phase when completing sessions with client A, who exhibited aggression during some sessions (sessions 16–18 and 22–24). To control for the potential confounding effect of aggression on procedural fidelity and interobserver agreement, and ensure the validity of the outcomes for client A’s sociability assessments, Margot completed the remaining child sessions (sessions 25–30) with client B. The right panel of Fig. 3 shows the interobserver agreement data for each participant across sessions. Most participants (Liz, Erica, and Margot) exhibited increases or stable high interobserver agreement during all phases. Vivian exhibited stable high interobserver agreement in the duration condition, and lower and variable interobserver agreement in the momentary time sampling condition. Margot exhibited temporary decreases during the reversal to child phase when completing sessions with client A. Some participants (e.g., Liz) exhibited lower procedural fidelity or interobserver agreement across conditions during initial sessions, but performance improved across subsequent sessions. These improvements may be attributable to repeated exposure to or practice implementing the procedures while collecting data. Child sociability outcomes across participants as recorded by a trained observer are available as Supporting Information C.

Figure 4 shows the mean procedural fidelity (left panel) and interobserver agreement (right panel) for each condition. Mean procedural fidelity scores remained relatively high across conditions and phases, especially during role-play sessions. During child sessions, we obtained slightly higher procedural fidelity during the duration condition and more variability across participants was evident. Mean interobserver agreement scores also remained relatively high across conditions and phases and no clear differences across phases were evident. Considering data from all phases, three participants yielded slightly higher interobserver agreement during the momentary time sampling condition, whereas one participant yielded much higher interobserver agreement during the duration condition. Overall, procedural fidelity and interobserver agreement were relatively high across conditions for at least three of four participants.

Table 4 depicts all participant data collection preferences and feasibility ratings for conducting sociability assessments with a data collection requirement. Participants reported mixed preferences for a concurrent data collection requirement while conducting sociability assessments. Margot preferred no live data collection, Erica preferred momentary time sampling, and both Liz and Vivian preferred duration. On a scale of 1 to 5, the mean feasibility ratings were 1.25 (range, 1 to 2) for no live data collection, 2.5 (range, 1 to 4) for momentary time sampling, and 2.5 (range, 2 to 3) for duration. Vivian preferred duration data collection and exhibited higher interobserver agreement during that condition, showing correspondence between preference and performance. Correspondence cannot be examined for other participants, as they did not exhibit differentiated performance between data collection methods.

**Table 4:** Study 2 participants’ data collection preference and feasibility ratings

Participants	Preferred data collection method	Rating		
		No data	MTS	Duration
Liz	Duration	1	2	2
Erica	MTS	1	1	3
Margot	No Data	1	4	3
Vivian	Duration	2	3	2

*MTS* Momentary time sampling

## General Discussion

The purpose of the current studies was to evaluate the feasibility of collecting live discontinuous and continuous data while implementing sociability assessments. Specifically, we evaluated the feasibility of collecting momentary time sampling and duration data while implementing sociability assessment procedures with a role-play partner (both studies) or child on the autism spectrum (study 2 only). The results of study 1 suggest that (a) participants could collect accurate momentary time sampling data while implementing the assessment with similar procedural fidelity as when no data collection was required and that (b) requiring implementers to collect duration data may slightly decrease procedural fidelity, although it is unclear if these differences are clinically significant (i.e., would alter sociability assessment outcomes). Interobserver agreement remained relatively high across conditions. We obtained slightly higher scores for momentary time sampling than duration for most participants, but interobserver agreement was higher for duration for two out of seven participants.

Study 2 included child sessions, improved training prior to the evaluation, and digital momentary time sampling. Results of study 2 suggest that high levels of procedural fidelity can be achieved while collecting momentary time sampling or duration data during sessions conducted with a role-play partner or child. For most participants, we obtained similar, high levels of interobserver agreement for momentary time sampling and duration across role-play and child sessions, but one participant obtained much lower interobserver agreement for momentary time sampling. In part, we may have obtained these differences because a single error impacts interobserver agreement more for momentary time sampling (i.e., 16 opportunities for agreement) than for duration (i.e., 48 opportunities for agreement).

Across both studies, participants’ preferred method varied between collecting no data, momentary time sampling, or duration data. Generally, participants rated no data collection as more feasible than momentary time sampling or duration. Participants rated momentary time sampling and duration as similarly feasible, with most assigning both a rating of either 2 or 3 on scale of 1 (i.e., very easy/feasible) to 5 (i.e., very difficult/infeasible). One aspect that may have prevented momentary time sampling from being more feasible than duration is that participants were required to attend to a timer to determine when each 30-s interval elapsed, which may have increased difficulty relative to formats in which the end of the interval is signaled by an auditory or tactile prompt (e.g., Gunter et al., 2003; Seward & Redner, 2024). Overall, results from both studies indicate that it may be feasible to maintain relatively high procedural fidelity and interobserver agreement when collecting live discontinuous or continuous data while concurrently implementing sociability assessment procedures. Additionally, there was correspondence between the data collection method that was most preferred and the data collection method with the highest performance for some participants (e.g., Lonnie and Vivian), but not others (e.g., Victoria). It may be useful for future research to further investigate the relation between preference and correspondence, especially as performance improves with repeated practice (e.g., Bacotti et al., 2021; Thibodeaux et al., 2025).

The current study extends previous research by evaluating the feasibility of different methods of data collection *while implementing* assessment procedures. Researchers have evaluated the feasibility of methods of data collection when it was the only response requirement (Hanley et al., 2007; Kolt & Rapp, 2014) and even systematically manipulated the difficulty of data collection (e.g., Rolider et al., 2012). However, to our knowledge, the current study is the first to evaluate the feasibility of different methods of data collection while implementing assessment procedures. In clinical practice, behavior analysts may often serve the dual role of implementer and data collector (e.g. Fiske & Delmolino, 2012). Therefore, further evaluating the feasibility of methods of data collection with the concurrent requirements of conducting sociability assessments and expanding this type of evaluation beyond sociability assessments are important directions for future research. Our findings suggest that implementers can conduct sociability assessments with high procedural fidelity while concurrently collecting accurate momentary time sampling or duration data.

Although previous research on sociability assessments suggest they may require additional personnel or non-assessment time to conduct, the current studies demonstrate that, following substantial training, a single individual can readily serve the dual role of implementer and data collector. Therefore, additional personnel or non-assessment time are not required and need not hinder sociability assessments from being routinely conducted in research or clinical practice. Both momentary time sampling and duration may allow for similar conclusions about the function of social interaction (see Supporting Information A), so behavior analysts may prioritize their data collection preference or default to collecting duration data, which may allow for more precise conclusions and could facilitate within-session analyses (Morris & Pizzuto, 2023). However, an interesting direction for future research would be to evaluate methods of collecting data on other clinically relevant participant behaviors during sociability assessments (e.g., Morris & Vollmer, 2022a), which may be more feasible in addition to momentary time sampling than duration data collection.

The results of both studies replicate the findings of previous research evaluating the feasibility of using discontinuous methods of data collection: (a) momentary time sampling can yield high levels of interobserver agreement and (b) methods that are rated as feasible or preferred by data collectors can readily be identified (Hanley et al., 2007; Kolt & Rapp, 2014). Whereas previous research has focused on comparing the feasibility of and preference for different interval sizes (Hanley et al., 2007) or different interval sizes and different methods of discontinuous measurement (Kolt & Rapp, 2014), the current study extends this literature by including a continuous method of data collection as a point of comparison. Many participants obtained high interobserver agreement and provided similar feasibility ratings across the continuous and discontinuous methods evaluated.

The current study has several limitations with corresponding directions for future research. First, our participants were undergraduate students with little to no experience providing behavior analytic services. It remains unclear whether our results would generalize to students with other demographic backgrounds, registered behavior technicians with extensive experience, or practicing, masters-level, board-certified behavior analysts. Replicating the current study with these populations is an important direction for future research.

Second, we somewhat arbitrarily selected 30 s as the interval to be used in the evaluations of momentary time sampling. Our results suggest that momentary time sampling with 30-s intervals may allow for similar conclusions as duration data (see Supporting Information A) and may be feasible to use while implementing sociability assessment procedures. However, smaller intervals could further improve correspondence with duration, whereas larger intervals could further improve feasibility. Future research should parametrically evaluate the effects of different momentary time sampling intervals on correspondence, feasibility, and preference.

Third, aspects of our training, role-play sessions (i.e., rate of switching, type of prompting), and data collection systems varied unsystematically between studies 1 and 2. These unsystematic procedural variations preclude clear conclusions about why slightly different results may have been obtained across studies and the impact of these procedural variations will be important to explore in future research. For example, systematic, within-participant manipulation of the modality of data collection (e.g., digital versus paper and pen) and the characteristics of role-play partner behavior (e.g., high versus low rate of switching) may elucidate variables that influence procedural fidelity, interobserver agreement, and preference.

Fourth, disagreements may have differentially impacted interobserver agreement for momentary time sampling in comparison to duration. As mentioned above, there were only 16 opportunities for agreement with momentary time sampling (i.e., 30-s intervals) whereas there were 48 opportunities for agreement with duration (i.e., 10-s intervals), meaning the extent to which each disagreement decreased interobserver agreement varied across the data collection methods. It is possible that interobserver agreement scores were deflated for momentary time sampling due to the smaller number of opportunities for agreement. We used 10-s intervals in evaluating interobserver agreement for duration data to remain consistent with previous research (e.g., Morris & Vollmer, 2021). However, in future research it may be useful to attempt to equate the interobserver agreement calculations across data collection methods, potentially by using 30-s bins and interval-by-interval agreement in evaluating interobserver agreement for duration data.

Finally, participants’ preference and feasibility ratings often did not correspond to their procedural fidelity and interobserver agreement data. This type of disconnect between preference and efficacy (e.g., Goldman et al., 2025; Morris et al., 2024) or indirect versus direct measures of preference (e.g., Ferguson et al., 2019; Hanley, 2010; Morris & Vollmer, 2022c) is not necessarily surprising, but nevertheless limits the conclusions that can be drawn about preference and feasibility. In future research, it could be useful to incorporate a more formal evaluation of preference and social validity by incorporating a concurrent chains choice phase (e.g., Auten et al., 2024; Linton et al., 2025) in which participants select the method of data collection they will implement in the upcoming session. Additionally, our preference questionnaire described the no data collection condition as collecting retrospective data after sessions occurred. This isolated preference for implementing sociability assessments with or without concurrent data collection requirements and ensured that whether or not any data collection was required could not exert a confounding influence on preference. However, participants were not required to collect data from video recordings following no data collection sessions during the recording feasibility evaluation. Future research on simultaneous data collection and implementation may evaluate the no live data collection condition with a retrospective data collection requirement to further mirror research and clinical practice.

The current studies constitute important steps toward improving the feasibility and adoptability of sociability assessments. Previous research has focused on reducing assessment complexity (e.g., Morris & Vollmer, 2021) as well as the amount of time (e.g., Morris & Vollmer, 2020c) and number of sessions (e.g., Morris & Pizzuto, 2023) required to draw conclusions about the function of social interaction, but the current study is the first to directly evaluate the feasibility of conducting sociability assessments. The trajectory of this line of research parallels the increasing focus on effectiveness, feasibility, and adoptability that can be seen in other areas (e.g., Ghaemmaghami et al., 2021). However, rather than progressing from efficacy to effectiveness, more research is needed in both domains (Morris & Pizzuto, 2023). Future research should continue to explore methods of improving the efficacy and precision with which sociability assessments delineate the function of social interaction while also continuing to improve the effectiveness, feasibility, and adoptability of sociability assessments for routine use in research and practice. Beyond the nascent literature on sociability assessment, the methods employed in the current studies also have the potential to impact other areas of research. Evaluating the feasibility of implementing procedures with concurrent data collection requirements has the potential to improve the adoptability and dissemination of myriad behavior-analytic assessment or intervention strategies and could facilitate progress toward closing the research-to-practice gap.

## Supplementary Information

Below is the link to the electronic supplementary material.

Supplementary file1 (DOCX 543 KB)

Supplementary file2 (XLSX 19 KB)

## Data Availability

Data that support the findings of this study are available online as supplementary material. Additional data are available from the corresponding author upon reasonable request.

## Footnotes

1 Despite evidence suggesting these discontinuous measures may approximate continuous measures such as duration, it is worth noting that partial interval recording tends to systematically overestimate, whereas momentary time sampling introduces unsystematic error (Meany-Daboul et al., 2007).

2 Published sociability assessment research refers to the “independent play” side as the “alone” side. We opted to use the term “independent play” side because an adult is always present in the room.

3 It is important to note that previous research (e.g., Morris & Vollmer, 2020c; 2021) has recorded and reinforced mands for the therapist movement (e.g., “Leave me alone” or “Come play with me”), but these were neither displayed by role-play partners nor observed with child participants during the current study.

## Declarations

All procedures involving human participants were performed in accordance with the ethical standards of the institutional and/or national research committee and with the 1964 Helsinki declaration and its later amendments or comparable ethical standards. All procedures were approved by the Institutional Review Boards for Louisiana State University or the University of Miami.

### Author Note

G.E.S. and G.S. implemented study procedures, collected, analyzed, and graphed data, contributed to the initial manuscript draft, and contributed to editing and revision of the manuscript. J.B. and S.M. conceptualized and designed the study, provided supervision and resources throughout the research process, contributed to the initial manuscript draft, and contributed to editing and revision of the manuscript. S.M. also collected, analyzed, and graphed data. Correspondence regarding this manuscript should be addressed to Samuel L. Morris, Louisiana State University, 236 Audubon Hall, Baton Rouge, LA, 70803, USA

## References

[CR1] Agana, T. R. T., Sidener, T. M., Rodriguez, N. M., Reeve, S. A., & Pane, H. M. (2025). An analysis of the generalization of pretend play from real objects to toys. *Journal of Applied Behavior Analysis*. 10.1002/jaba.70017 PMC12302316 40493445

[CR2] Alvero, A. M., Struss, K., & Rappaport, E. (2008). Measuring safety performance: A comparison of whole, partial, and momentary time-sampling recording methods. *Journal of Organizational Behavior Management,**27*(4), 1–28. 10.1300/J075v27n04_01

[CR3] Auten, E. M., Van Camp, C., & Ferguson, A. B. (2024). A review of the concurrent-chains arrangement to assess intervention choice: 2018–2023. *Journal of Applied Behavior Analysis,**57*(2), 319–330. 10.1002/jaba.1059 38299638

[CR4] Bacotti, J. K., Grauerholz, F. E., Morris, S. L., & Vollmer, T. R. (2021). Identifying the relation between feedback preferences and performance. *Journal of Applied Behavior Analysis,**54*(2), 668–683. 10.1002/jaba.804 33440028

[CR5] Behavior Analyst Certification Board. (2018). *Supervisor training curriculum outline: (2.0)*. Littleton, CO: Author. Retrieved from: https://www.bacb.com/wp-content/uploads/2022/01/Supervision_Training_Curriculum_240206-a.pdf

[CR6] Call, N. A., Shillingsburg, M. A., Bowen, C. N., Reavis, A. R., & Findley, A. J. (2013). Direct assessment of preferences for social interactions in children with autism. *Journal of Applied Behavior Analysis,**46*(2), 821–826. 10.1002/jaba.692 24114289

[CR7] Cengher, M., Clayborne, J. C., & O’Connor, J. T. (2021). Assessment and treatment of escape from attention in the form of conversation. *Behavioral Interventions,**36*(1), 21–39. 10.1002/bin.1754

[CR8] Cengher, M., O’Connor, J. T., & Strohmeier, C. W. (2022). Assessment and treatment of problem behavior maintained by escape from attention. *Behavioral Interventions,**37*(2), 204–221. 10.1002/bin.1838

[CR9] Clay, C. J., Samaha, A. L., Bloom, S. E., Bogoev, B. K., & Boyle, M. A. (2013). Assessing preference for social interactions. *Research in Developmental Disabilities,**34*(1), 362–371. 10.1016/j.ridd.2012.07.028 23009945

[CR10] Devine, S. L., Rapp, J. T., Testa, J. R., Henrickson, M. L., & Schnerch, G. (2011). Detecting changes in simulated events using partial-interval recording and momentary time sampling iii: Evaluating sensitivity as a function of session length. *Behavioral Interventions,**26*(2), 103–124. 10.1002/bin.328

[CR11] Dudley, L. L., Axe, J. B., Allen, R. F., & Sweeney-Kerwin, E. J. (2019). Establishing praise as a conditioned reinforcer: Pairing with one versus multiple reinforcers. *Behavioral Interventions,**34*(4), 534–552. 10.1002/bin.1690

[CR12] Ferguson, J. L., Cihon, J. H., Leaf, J. B., Van Meter, S. M., McEachin, J., & Leaf, R. (2019). Assessment of social validity trends in the journal of applied behavior analysis. *European Journal of Behavior Analysis,**20*(1), 146–157. 10.1080/15021149.2018.1534771

[CR13] Fiske, K., & Delmolino, L. (2012). Use of discontinuous methods of data collection in behavioral intervention: Guidelines for practitioners. *Behavior Analysis in Practice,**5*(2), 77–81. 10.1007/BF03391826 PMC3592492 23730469

[CR14] Gardenier, N. C., MacDonald, R., & Green, G. (2004). Comparison of direct observational methods for measuring stereotypic behavior in children with autism spectrum disorders. *Research in Developmental Disabilities,**25*(2), 99–118. 10.1016/j.ridd.2003.05.004 15026089

[CR15] Ghaemmaghami, M., Hanley, G. P., & Jessel, J. (2021). Functional communication training: From efficacy to effectiveness. *Journal of Applied Behavior Analysis,**54*(1), 122–143. 10.1002/jaba.762 32929757

[CR16] Goldman, K. J., Martinez, C., Hack, G. O., Hernandez, R., Laureano, B., Argueta, T., Sams, R., & DeLeon, I. G. (2025). Correspondence between preference for and efficacy of behavioral interventions: A systematic review. *Journal of Applied Behavior Analysis, 58*(1), 118–133. 10.1002/jaba.2924 39567258

[CR17] Gunter, P. L., Venn, M. L., Patrick, J., Miller, K. A., & Kelly, L. (2003). Efficacy of using momentary time samples to determine on-task behavior of students with emotional/behavioral disorders. *Education and Treatment of Children,**26*(4), 400–412.

[CR18] Hanley, G. P. (2010). Toward effective and preferred programming: A case for the objective measurement of social validity with recipients of behavior-change programs. *Behavior Analysis in Practice,**3*(1), 13–21. 10.1007/BF03391754 PMC3004679 22479668

[CR19] Hanley, G. P., Cammilleri, A. P., Tiger, J. H., & Ingvarsson, E. T. (2007). A method for describing preschooler’s activity preferences. *Journal of Applied Behavior Analysis,**40*(4), 603–618. 10.1901/jaba.2007.603-618 PMC2078569 18189093

[CR20] Harper, J. M., Iwata, B. A., & Camp, E. M. (2013). Assessment and treatment of social avoidance. *Journal of Applied Behavior Analysis,**46*(1), 147–160. 10.1002/jaba.18 24114091

[CR21] Harrop, A., & Daniels, M. (1986). Methods of time sampling: A reappraisal of momentary time sampling and partial interval recording. *Journal of Applied Behavior Analysis,**19*(1), 73–77. 10.1901/jaba.1986.19-73 PMC1308042 16795692

[CR22] Iwata, B. A., Dorsey, M. F., Slifer, K. J., Bauman, K. E., & Richman, G. S. (1982). Toward a functional analysis of self-injury. *Analysis and Intervention in Developmental Disabilities,**2*(1), 3–20. 10.1016/0270-4684(82)90003-9

[CR23] Jimenez-Gomez, C., Hannula, C., Liggett, A. P., Shvarts, S., & Podlesnik, C. A. (2024). Evaluating functions of praise for children diagnosed with autism spectrum disorder. *Journal of Applied Behavior Analysis,**57*(3), 584–598. 10.1002/jaba.1079598 38709201

[CR24] Kelly, M. A., Roscoe, E. M., Hanley, G. P., & Schlichenmeyer, K. (2014). Evaluation of assessment methods for identifying social reinforcers. *Journal of Applied Behavior Analysis,**47*(1), 113–135. 10.1002/jaba.107 24604393

[CR25] Kodak, T., Northup, J., & Kelley, M. E. (2007). An evaluation of the types of attention that maintain problem behavior. *Journal of Applied Behavior Analysis,**40*(1), 167–171. 10.1901/jaba.2007.43-06 PMC1868812 17471800

[CR26] Kolt, L. D., & Rapp, J. T. (2014). Assessment of therapists’ preferences for discontinuous measurement systems. *Behavioral Interventions,**29*(4), 304–314. 10.1002/bin.1392

[CR27] LeBlanc, L. A., Lund, C., Kooken, C., Lund, J. B., & Fisher, W. W. (2020). Procedures and accuracy of discontinuous measurement of problem behavior in common practice of applied behavior analysis. *Behavior Analysis in Practice,**13*(2), 411–420. 10.1007/s40617-019-00361-6 PMC7314895 32642397

[CR28] LeBlanc, L. A., Raetz, P. B., Sellers, T. P., & Carr, J. E. (2015). A proposed model for selecting measurement procedures for the assessment and treatment of problem behavior. *Behavior Analysis in Practice,**9*(1), 77–83. 10.1007/s40617-015-0063-2 PMC4788644 27606232

[CR29] Linton, E., Gomes, G., & Donaldson, J. M. (2025). Effects of differential reinforcement and time-out on the unsafe playground behavior of young children. *Journal of Applied Behavior Analysis*. 10.1002/jaba.70009 40114598

[CR30] Mattson, S. L., Higbee, T. S., Campbell, V. E., Lindgren, N. A., Osos, J. A., & Nichols, B. (2023). Using generic picture cues to promote verbal initiations during play. *Journal of Applied Behavior Analysis,**56*(4), 816–830. 10.1002/jaba.1014 37646600

[CR31] Meany-Daboul, M. G., Roscoe, E. M., Bourret, J. C., & Ahearn, W. H. (2007). A comparison of momentary time sampling and partial-interval recording for evaluating functional relations. *Journal of Applied Behavior Analysis,**40*(3), 501–514. 10.1901/jaba.2007.40-501 PMC1986695 17970263

[CR32] Morris, C., Zarcaro, E. A., Perrin, J., & Ellsworth, M. E. (2024). Discrepancies between treatment preference and effectiveness. *Behavioral Interventions,**39*(4), Article e2057. 10.1002/bin.2057

[CR33] Morris, S. L., Gallagher, M. L., & Allen, A. E. (2023). A review of methods of assessing preference for social stimuli. *Journal of Applied Behavior Analysis, 56*(2), 416–427. 10.1002/jaba.981 36922701

[CR34] Morris, S. L., Call, N. A., Lomas Mevers, J., & Taylor, P. M. (2025). A behavioral economic measure of sensitivity to social rewards in children with and without autism spectrum disorder. *Research In Autism,**127*, Article 202663. 10.1016/j.reia.2025.202663 PMC13521615 42663006

[CR35] Morris, S. L., & Pizzuto, A. (2023). Secondary and within-session analyses to improve the feasibility and precision of assessments of sociability. *Journal of Applied Behavior Analysis,**56*(4), 853–868. 10.1002/jaba.1021 37665333

[CR36] Morris, S. L., & Vollmer, T. R. (2019). Assessing preference for different types of social interaction. *Journal of Applied Behavior Analysis,**52*(4), 1064–1075. 10.1002/jaba.597 31268168

[CR37] Morris, S. L., & Vollmer, T. R. (2020a). A comparison of methods for assessing preference for social interactions. *Journal of Applied Behavior Analysis,**53*(2), 918–937. 10.1002/jaba.692 32141096

[CR38] Morris, S. L., & Vollmer, T. R. (2020b). A comparison of picture and GIF-based preference assessments for social interaction. *Journal of Applied Behavior Analysis,**53*(3), 1452–1465. 10.1002/jaba.680 31965577

[CR39] Morris, S. L., & Vollmer, T. R. (2020c). Evaluating the function of social interaction using time allocation as a dependent measure: A replication and extension. *Journal of Applied Behavior Analysis,**53*(4), 2405–2420. 10.1002/jaba.750 32691893

[CR40] Morris, S. L., & Vollmer, T. R. (2021). Evaluating the function of social interaction for children with autism. *Journal of Applied Behavior Analysis,**54*(4), 1456–1467. 10.1002/jaba.850 34028027

[CR41] Morris, S. L., & Vollmer, T. R. (2022a). Increasing social time allocation and concomitant effects on mands, item engagement, and rigid or repetitive behaviors. *Journal of Applied Behavior Analysis,**55*(3), 814–831. 10.1002/jaba.919 35388493

[CR42] Morris, S. L., & Vollmer, T. R. (2022b). The matching law provides a quantitative description of social time allocation in children with autism. *Journal of Applied Behavior Analysis,**55*(3), 934–957. 10.1002/jaba.934 35607852

[CR43] Morris, S. L., & Vollmer, T. R. (2022c). Evaluating the validity of reported hierarchies of social reinforcers. *Behavior Analysis Research and Practice,**22*(4), 354–367. 10.1037/bar0000257

[CR44] Nuernberger, J. E., Smith, C. A., Czapar, K. N., & Klatt, K. P. (2012). Assessing preference for social interaction in children diagnosed with autism. *Behavioral Interventions,**27*(1), 33–44. 10.1002/bin.1336

[CR45] Pane, H. M., Sidener, T. M., Reeve, S. A., Kisamore, A., & Nirgudkar, A. (2022). A comparison of development-matched and age-matched targets on play skills of children with autism spectrum disorder. *Journal of Applied Behavior Analysis,**55*(1), 195–213. 10.1002/jaba.891 34806181

[CR46] Parsons, M. B., Rollyson, J. H., & Reid, D. H. (2012). Evidence-based staff training: A guide for practitioners. *Behavior Analysis in Practice,**5*(2), 2–11. 10.1007/BF03391819 PMC3592486 23730462

[CR47] Piazza, C. C., Bowman, L. G., Contrucci, S. A., Delia, M. D., Adelinis, J. D., & Goh, H. L. (1999). An evaluation of the properties of attention as reinforcement for destructive and appropriate behavior. *Journal of Applied Behavior Analysis,**32*(4), 437–449. 10.1901/jaba.1999.32-437 PMC1284207 10641299

[CR48] Rapp, J. T., Colby, A. M., Vollmer, T. R., Roane, H. S., Lomas, J., & Britton, L. N. (2007). Interval recording for duration events: A re-evaluation. *Behavioral Interventions,**22*(4), 319–345.

[CR49] Rapp, J. T., Colby-Dirksen, A. M., Michalski, D. N., Carroll, R. A., & Lindenberg, A. M. (2008). Detecting changes in simulated events using partial-interval recording and momentary time sampling. *Behavioral Interventions: Theory & Practice in Residential & Community-Based Clinical Programs,**23*(4), 237–269.

[CR50] Rolider, N. U., Iwata, B. A., & Bullock, C. E. (2012). Influences of response rate and distribution on the calculation of interobserver reliability scores. *Journal of Applied Behavior Analysis,**45*(4), 753–762. 10.1901/jaba.2012.45-753 PMC3545499 23322930

[CR51] Sainsbury, E. L., Sidener, T. M., Taylor-Santa, C., Reeve, K. F., & Sidener, D. W. (2024). Evaluation of a discrimination training procedure for establishing praise as a reinforcer. *Journal of Applied Behavior Analysis,**57*(3), 776–783. 10.1002/jaba.1071 38501539

[CR52] Schmidt, M. G., Rapp, J. T., Novotny, M. A., & Lood, E. A. (2013). Detecting changes in non-simulated events using partial interval recording and momentary time sampling: Evaluating false positives, false negatives, and trending. *Behavioral Interventions,**28*(1), 58–81. 10.1002/bin.1354

[CR53] Seaver, J. P., & Bourret, J. C. (2020). Producing mands in concurrent operant environments. *Journal of Applied Behavior Analysis,**53*(1), 366–384. 10.1002/jaba.592 31219193

[CR54] Seward, R., & Redner, R. (2024). A replication of extended implementation and social interaction during the Step it UP! game for adults with disabilities. *Journal of Applied Behavior Analysis,**57*(4), 1042–1057. 10.1002/jaba.1098 38963347

[CR55] Sidman, M. (1960). *Tactics of scientific research: Evaluating experimental data in psychology*. Basic Books.

[CR56] Sivaraman, M., & Fahmie, T. A. (2018). Using common interests to increase socialization between children with autism and their peers. *Research in Autism Spectrum Disorders,**51*(1), 1–8. 10.1016/j.rasd.2018.03.007

[CR57] Slanzi, C. M., & Fernand, J. K. (2024). On the use and benefits of electronic data collection systems: A tutorial on Countee. *Behavior Analysis in Practice*. 10.1007/s40617-024-00964-8 PMC11707102 39790922

[CR58] Slocum, S. K., Scheithauer, M., & Muething, C. (2021). Demonstration of a negative reinforcement preference assessment to determine aversiveness of types of social interaction. *Behavioral Interventions,**37*(3), 611–625. 10.1002/bin.1844

[CR59] Smaby, K., MacDonald, R. P. F., Ahearn, W. H., & Dube, W. V. (2007). Assessment protocol for identifying preferred social consequences. *Behavioral Interventions,**22*(4), 311–318. 10.1002/bin.242

[CR60] Thibodeaux, J. J., Taylor, P. M., Bacotti, J. K., & Morris, S. L. (2025). Evaluating feedback frequency preference and its relation to task performance. *Journal of Applied Behavior Analysis,**58*(1), 232–242. 10.1002/jaba.2932 39722543

[CR61] Vollmer, T. R., Sloman, K. N., & St. Peter Pipkin, C. (2008). Practical implications of data reliability and treatment fidelity monitoring. *Behavior Analysis in Practice,* 1, 4–11. 10.1007/BF03391722 PMC2846587 22477682

