# Supplementary material for: Evaluating the Feasibility of Methods of Live Data Collection for Sociability Assessments
Source: Behav Anal Pract. 2025 Oct 9;19(3):1342–60. doi: 10.1007/s40617-025-01101-9 (PMC13638883; doi:10.1007/s40617-025-01101-9)
Supplement: Supplementary file 1 [file 40617_2025_1101_MOESM1_ESM.docx]

**Supporting Information A**

**Evaluating Correspondence Between Discontinuous and Continuous Measures**

**Method**

***Participants, Setting, and Materials***

This study involved a retrospective re-analysis of data originally published in Morris and Vollmer (2021). Morris and Vollmer conducted sociability assessments with 21 children diagnosed with autism spectrum disorder who were receiving center-based, behavior-analytic services. Duration on the social side was the primary dependent measure. We selected 8 participants’ data for re-analysis: three participants that responded socially (Lance, Beckham, and Booker), three participants that responded indifferently (Chloe, Felicity, and Jessica), and two participants that responded avoidantly (Edward and Cedric). As described below, we re-analyzed these participant’s duration data to evaluate their degree of correspondence with data produced via partial interval recording, whole interval recording, and momentary time sampling using 30-s intervals. We selected 30-s intervals based on the assumption that this duration may be larger enough to increase the feasibility of collecting data while implementing sociability assessments, but small enough to maintain a high degree of correspondence with duration data.

***Dependent Measures and Interobserver Agreement***

We reviewed the duration of social time allocation data collected by trained observers in Morris and Vollmer (2021) according to the response definitions described in the General Method section. These trained observers collected data using Countee® during live or video-recorded sessions. We re-analyzed the duration data by dividing the Countee® second-by-second data stream for each session into 30-s intervals and constructing an excel spreadsheet that was automated to implement rules consistent with partial interval recording, whole interval recording, and momentary time sampling. For partial interval recording, the spreadsheet scored social side for a given interval if the duration data indicated the participant was on the social side for any portion (i.e., even 1 s) of that interval. For whole interval recording, the excel spreadsheet scored social side for a given interval if the duration data indicated the participant was on the social side for the whole (i.e., entire 30-s) interval. For momentary time sampling, the excel spreadsheet scored social side for a given interval if the duration data indicated the participant was on the social side during the last second of that interval, regardless of how they allocated their time for the rest of the interval.

As reported in Morris and Vollmer (2021), a second, independent observer collected duration data for 43.8% of all sessions and at least 25% of each individual participant’s assessment sessions. Proportional interobserver agreement was calculated by dividing the session into 10-s intervals and comparing each observer’s data on an interval-by-interval basis. Within each interval, the smaller number of seconds on the social side was divided by the larger number of seconds on the social side to obtain a proportion. The sum of the proportions for each interval was divided by the total number of intervals and then multiplied by 100. Across participants, mean interobserver agreement was 96.2% (range, 94% to 100%).

***Data Analysis***

We calculated the *percentage of session on the social side* by dividing the duration the participant was on the social side by the total session duration. For each method of discontinuous measurement, we calculated the *percentage of intervals on the social side* by dividing the number of intervals the participant was scored as being on the social side by the total intervals in a session. We compared continuous (i.e., duration) and discontinuous (partial-interval recording, whole-interval recording, momentary time sampling) measures on a session-by-session basis for each participant. We evaluated correspondence between the different types of measurement in two ways. First, we graphed each participant’s assessment results based on percentage of session on the social side (i.e., duration) as well as percentage of intervals on the social side according to whole-interval recording, partial-interval recording, and momentary time sampling in order to evaluate the degree to which each type of measurement allowed for similar conclusions about sociability based on visual analysis. Second, we calculated the difference between the percentage of session on the social side as well as the percentage of intervals on the social side according to whole-interval recording, partial-interval recording, and momentary time sampling for each individual assessment session. This analysis summarized the degree of correspondence and types of differences obtained for each method of discontinuous measurement and facilitated comparisons within and across participants with varied patterns of responding (e.g., social, indifferent, or avoidant). We calculated the difference by subtracting duration measure (i.e., percentage of session on the social side) from each of the three methods of discontinuous measurement (i.e., percentage of intervals on the social side for whole-interval recording, partial-interval recording, and momentary time sampling) for each session. Difference values above zero indicate an overestimate of social time allocation (i.e., the discontinuous measure was higher or exceeded the duration measure). Difference values below zero indicate an underestimate of social time allocation (i.e., the discontinuous measure was lower than the duration measure). Difference values of zero indicate no difference between the scoring produced by the discontinuous measure and duration measure for social time allocation (i.e., perfect correspondence).

**Results**

Figure SA 1 shows the difference in social time allocation according to each method of discontinuous measurement. Participants are displayed on the x-axis and the difference between each method of discontinuous measurement and the duration on the social side is displayed on the y-axis. Participants are ordered from the highest (left) to lowest (right) mean duration on the social side. Each data point displays the difference in social time allocation for a given session. Horizontal black bars display the mean difference between a given discontinuous measurement method and duration for a given participant. Results were differentiated between the three methods of discontinuous measurement. Partial interval recording consistently produced overestimates (i.e., individual session and mean differences above 0) and whole interval recording consistently produced underestimates (i.e., individual session and mean differences below 0) of social time allocation when compared to the duration data. Momentary time sampling produced slight or no differences (i.e., individual session and mean differences slightly above, slightly below, or at zero) when compared to the duration data. Therefore, momentary time sampling had the greatest degree of correspondence with the duration data, irrespective of the function of social interaction across participants. However, the direction of differences obtained for momentary time sampling varied slightly based on participants’ pattern of social time allocation. Mean differences above zero versus below zero were obtained with participants for whom social interactions functioned as reinforcing (Lance, Beckham, Booker) versus aversive stimuli (Cedric, Edward), respectively.

Figure SA 2 includes session-by-session data that displays the direction of differences obtained for each type of discontinuous measurement when compared to duration data corresponds with the overall outcomes described above. More specifically, differences between partial and whole interval data compared to duration data were sufficiently large that they may result in different conclusions about the function of social interaction for many of these participants. In contrast, there were minimal differences between momentary time sampling and duration data, which were sufficiently similar that they may allow for the same conclusions about the function of social interaction for these participants. On exception was Chloe, for whom momentary time sampling consistently overestimated duration on the social side and, therefore, may have indicated that social interaction was a reinforcing rather than a neutral stimulus.

**Figure SA 1**

*Difference in Percentage of Social Time Allocation Across Data Collection Methods*

**Figure SA 2**

*Timeseries Comparison of Different Measures of Social Time Allocation*

**Supporting Information B**

**Study 1 Pre-Experimental Procedures**

**Reading and Discussion**

The initial reading assignment occurred one week prior to the discussion. Participants read Morris and Vollmer (2021) to obtain some foundational knowledge about sociability assessments. We asked participants a variety of questions about the purpose, procedures, and analyses during an open-group discussion for approximately 1 hr. Participants also had the opportunity to ask questions.

**Data Collection Training**

The initial data collection training started on the same day as the discussion (i.e., the remaining 30 min of lab meeting) and then occurred twice a week for up to two weeks in 1.5-hr blocks. Two students with previous experience and training related to sociability assessments were also present and assisted with the training and discussion. Participants received definitions for duration on the social side, social switches, avoidant switches, and therapist movement (described in the General Method section). Participants viewed a video of a sociability assessment session while a trainer narrated what to record and how data collection varied depending on changes in participant behavior (e.g., when the child was on and to score social side). Participants only received training on using Countee® to collect duration on the social side and the frequency of social and avoidant switches. Then, participants scored two videos of sociability assessment sessions that differed in difficulty (i.e., amount of movement exhibited by the child in the assessment). Participants continued scoring each video until they achieved greater than 96% interobserver agreement with a trained observer. Participants received individualized feedback after each data submission (e.g., "it seems you're not capturing all of the switches" or "you're not turning off the duration counter quickly enough").

**Behavioral Skills Training**

A trainer reviewed the implementer behavior that occurred on the social and independent play sides and answered any questions before practicing implementing sociability assessments without collecting data. Next, the trainer modeled perfect implementation of sociability assessments while narrating how to provide social interactions, respond to clear and unclear mands, switch sides, and respond when the role-play partner was on the independent play side. Then, the trainer discussed common errors or deviations from the sociability assessment procedures and answered any questions. Finally, the participants rehearsed implementing sociability assessments with the trainer serving as a role-play partner, who provided immediate corrective feedback and praise on participant’s performance throughout the rehearsal. The participants practiced the implementation of sociability assessments with a role-play partner until they completed two rehearsal sessions with no more than two errors (i.e., minimum of 96% procedural integrity).

**Study 2 Pre-Experimental Procedures**

**Reading and Discussion**

We replicated the Study 1 article discussion format, except the initial reading assignment occurred 2.5 weeks prior to the discussion. Seven research assistants enrolled for undergraduate course credits or internship hours, the four participants, and three trainers were present. A trainer used a slideshow to facilitate the discussion and provide attendees with opportunities to ask and answer questions about the purpose, procedures, dependent measures, and findings (i.e., function of social interactions across participants) for approximately 30 min. Participants and the other attendees progressed to the data collection training for the second portion of lab meeting.

**Data Collection Training**

A trainer progressed through a slideshow that included operational definitions for dependent measures (described in the General Method section), videos of two sociability assessment sessions, and visual task analyses (screenshots) for setting up two Countee® templates that included frequency and duration keys for momentary time sampling and duration data collection, respectively. Attendees followed screenshots and instructions via the slideshow and a trainer to preset their Countee® templates for duration and momentary time sampling to 480 s and 510 s, respectively. Participants used mobile devices equipped with Countee® to practice the general functions of the application (e.g., pressing a key) while viewing a portion of one sociability assessment video during which a trainer described when to select a key, child behavior (i.e., social side, independent play side, switch), and implementer behavior (e.g., generic social interactions, errors). The instructional portion of the data collection training occurred for approximately 1 hr.

Then, participants progressed to independently scoring two videos of sociability assessment sessions that varied in difficulty (as described in Study 1) using momentary time sampling and duration templates on Countee® for the remainder of lab meeting (i.e., last 1.5 hr) and subsequent research shifts. Participants continued to score videos until they met greater than 96% proportional agreement for both duration and interval agreement for both momentary time sampling data submissions. Therefore, participants scored each video using momentary time sampling and duration data and uploaded their Countee® streams to an individual folder on a cloud-based server until they met criteria. Participants received feedback before their next data submission if interobserver agreement was below the 96% threshold for the same video two times consecutively. A trainer assessed interobserver agreement for and documented the number of data submissions for each participant. Table 4 shows the number of data submissions for each participant until they met the interobserver agreement criteria.

**Behavioral Skills Training**

We replicated the behavioral skills training procedures described in Study 1, which included reviewing sociability assessment procedures, answering questions, modeling correct implementation, discussing incorrect implementation, and rehearsing implementation with immediate praise and corrective feedback and delayed feedback following performance. A trainer assessed procedural integrity for and documented the number of rehearsals for each participant until they implemented two rehearsals without errors. Table 4 shows the number of rehearsals for each participant until they met criteria to advance to the sociability recording feasibility evaluation.

**Study 2 Data Collection and Behavioral Skills Training Results**

**Data Collection and Behavioral Skills Training**

Table 4 shows a summary of training outcomes that preceded the sociability recording feasibility evaluation. Participants met data collection training criteria within 7-12 submissions. The mean and ranges reported for each participant include all data collection training submission interobserver agreement scores. Participants only advanced to behavioral skills training after meeting the above 96% interobserver agreement criterion in their final submission for each video. Participants met behavioral skills training criteria within 3-4 rehearsals. Participants exhibited 1-4 errors per rehearsal before completing their last two rehearsals without errors.

*Summary of Study 2 Participant Training Outcomes*

|  | Number of Data Submissions | | | Number of Rehearsals |
| --- | --- | --- | --- | --- |
| Participants | Duration | MTS | Total | Total |
| Liz | 3 | 6 | 9 | 3 |
| Erica | 4 | 3 | 7 | 4 |
| Margot | 10 | 2 | 12 | 4 |
| Vivian | 8 | 3 | 11 | 3 |

*Note*. MTS = Momentary Time Sampling.

**Supporting Information C**

*Sociability Assessment Outcomes for Children Assessed in Study 2*

**Supporting Information D**

We calculated the percentage of procedural integrity errors that occurred on the social side by dividing the number of errors on the social side by the total errors across all child sessions for each participant and multiplied the quotient by 100. We calculated the percentage of procedural integrity errors on the independent play side by dividing the total errors on the independent play side by the total errors across all child sessions for each participant and multiplied the quotient by 100.

*Study 2 Percentage of Errors on the Social Side and Independent Play Sides Across All Participants*
